# Concentration of Mercury in the Livers of Small Terrestrial Rodents from Rural Areas in Poland

**DOI:** 10.3390/molecules24224108

**Published:** 2019-11-14

**Authors:** Maciej Durkalec, Agnieszka Nawrocka, Jacek Żmudzki, Aleksandra Filipek, Marcin Niemcewicz, Andrzej Posyniak

**Affiliations:** 1 Department of Pharmacology and Toxicology, National Veterinary Research Institute, Aleja Partyzantow 57, 24-100 Puławy, Poland; agnieszka.nawrocka@piwet.pulawy.pl (A.N.); aleksandra.filipek@piwet.pulawy.pl (A.F.); aposyn@piwet.pulawy.pl (A.P.); 2 Department of Swine Diseases; National Veterinary Research Institute, Aleja Partyzantow 57, 24-100 Puławy, Poland; jaca@piwet.pulawy.pl; 3 Biological Threats Identification and Countermeasure Centre, Military Institute of Hygiene and Epidemiology, Lubelska 2, 24-100 Puławy, Poland; marcinniem@wihe.pulawy.pl

**Keywords:** total mercury, liver, wild rodents, bank vole, common vole, yellow-necked mouse, striped field mouse

## Abstract

Small terrestrial mammals could be used as accumulative biomonitors of different environmental contaminants, but the knowledge of the level of Hg in their bodies is scant. The aim of our research was to verify the factors influencing Hg bioaccumulation and to analyze the concentration of total mercury (Hg) in the livers of four species of wild terrestrial rodents from different rural areas of Poland: the yellow-necked mouse (*Apodemus flavicollis*), striped field mouse (*Apodemus agrarius*), common vole (*Microtus arvalis*), and bank vole (*Myodes glareolus*). The concentration of total Hg was analyzed in liver tissue by atomic absorption spectrometry using a direct mercury analyzer. The concentration of Hg found in the livers of rodents ranged from <1 to 36.4 µg/kg of wet weight, differed between study sites, species, and sexes, and was related to body weight. We addressed feeding habits as potential causes of differences in liver Hg concentration among species.

## 1. Introduction

Mercury (Hg) is considered one of the most hazardous non-essential trace elements, and its fate in the environment, where it is ubiquitous, is a matter of concern worldwide [1]. The mechanisms of Hg toxicity are well known and depend on its chemical form [2,3]. Mercury can be emitted both from natural sources, such as volcanic eruptions and forest fires, and from anthropogenic sources, including coal combustion, notoriously used in the non-ferrous metals industry, cement production, and artisanal gold mining. Global anthropogenic Hg emissions were estimated at 2220 Mg in 2015 [4]. In Poland, anthropogenic emissions in 2016 were 10.3 Mg and were mainly caused by coal combustion for the production of electricity and heat, in industrial processing of non-ferrous metals, and in small household boilers [5,6]. The deposition of Hg may lead to contamination of both aquatic and terrestrial ecosystems [1], and inorganic Hg may be converted by microbial communities into more toxic methylmercury [7], which can be accumulated in the trophic chain [8]. Different organisms have been used as bioindicators of Hg pollution in terrestrial ecosystems, including invertebrates [9,10,11,12], birds [13,14,15], bats [16], shrews [17,18,19], moles [19], foxes [20], and mustelids [21,22]. Rodents are also considered good bioindicators of environmental pollution due to their widespread occurrence, high reproductive rate and abundance, short lifespan, and good availability [23,24]. However, differences in biotope preferences, feeding habits, and behavior may result in differential bioaccumulation of contaminants between particular species.

The bank vole (*Myodes glareolus*, Schreber 1780, formerly *Clethrionomys glareolus*) and the common vole (*Microtus arvalis*, Pallas 1778) belong to the *Arvicolinae* subfamily. The bank vole inhabits different types of woodlands [25]. Its diet is based mainly on aerial vegetative parts of plants and fruits but also includes invertebrates and fungi [26]. The common vole has a larger body weight than *M. glareolus* has (27.5 versus 17–20 g) [27,28], prefers open habitats, including meadows, pastures, and farming areas [29], and feeds mainly on herbaceous plants and grasses—invertebrates are very rarely present in its diet [26].

The striped field mouse (*Apodemus agrarius*, Pallas 1771) and yellow-necked mouse (*Apodemus flavicollis*, Melchior 1834) are two species that belong to the Muridae family and are also widely distributed in Eurasia, including Poland. The striped field mouse inhabits fields, meadows, gardens, the edges of forests, and roadside scrub parks [30] and is well adapted to urban habitats [31]. Its diet consists mainly of seeds, fruits, and invertebrates [26]. The yellow-necked mouse is considered a typical forest species and rarely occurs in urban areas [31]. The diet of *A. flavicollis* is more diverse compared to that of *A. agrarius*. Additionally to seeds, fruits, and invertebrates, *A. flavicollis* eats aboveground parts of plants, flowers, and fungi [26].

The objectives of our work were to analyze the concentration of total Hg in the livers of these four species of rodents and to verify the influence of study site, age, sex, and body weight (b.w.) on Hg bioaccumulation.

## 2. Results

**Figure 1:**
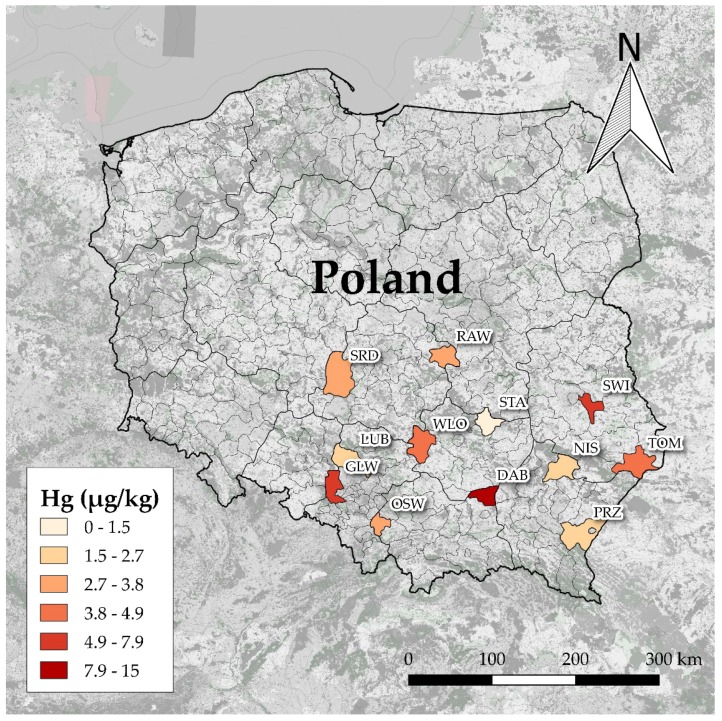
Concentrations of Hg in the livers of rodents from 12 study sites. The color scale represents estimated marginal means (in µg/kg of wet weight.). Results were averaged by species and sex.

**Figure 2:**
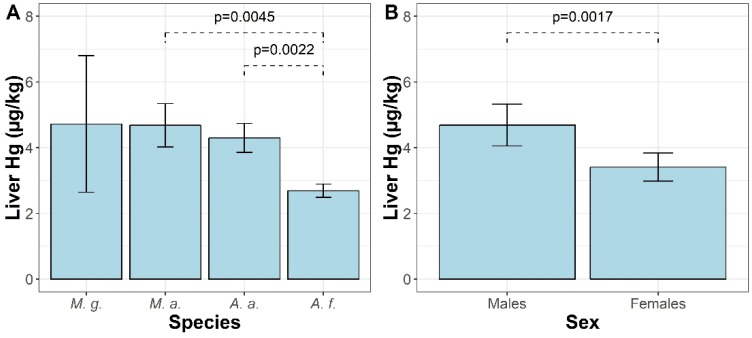
(**A**) Differences in Hg concentrations in the liver between species (*M. g*—*Myodes glareolus*; *M. a.*—*Microtus arvalis*; *A. a.*—*Apodemus agrarius*; *A. f.*—*Apodemus flavicollis*). Results (in µg/kg of wet weight) were averaged by study site and sex. (**B**) Differences in Hg concentrations in the liver between males and females (in µg/kg of wet weight). Results were averaged by study site and species. Both bar and whisker plots show estimated marginal means and standard errors that were back-transformed from the log scale. Differences between marginal means were verified on the log scale.

**Figure 3:**
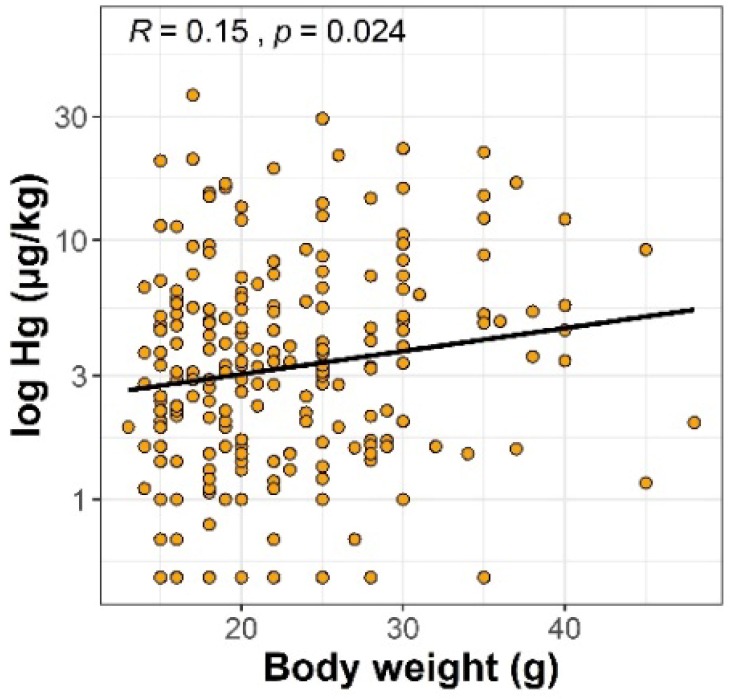
Spearman rank correlation coefficients between body weight of rodents and concentrations of Hg in their livers.

The concentration of Hg found in the livers of rodents ranged from <1 to 36.4 µg/kg wet weight. The descriptive statistics of Hg concentrations in the livers of rodents according to their species, sex, and sampling site are summarized in Supplementary Table S3. Generalized linear model (GLM) analysis showed that liver Hg concentrations were influenced by study site (F = 10.1, *p* = 2 × 10^−16^), sex (F = 9.9, *p* = 1.9 × 10^−3^), species (F = 6.1, *p* = 5.6 × 10^−4^), and body weight (F = 7.7, *p* = 5.9 × 10^−3^). Site-specific differences in liver Hg concentrations are shown in Figure 1. The highest estimated mean level of Hg in the livers of all species of rodents was at the DAB site (15 ± 4 μg/kg) and was higher than that found in other study sites (*p* < 0.05), with the exception of GLW and SWI. The second area with high liver Hg concentration was GLW (7.9 ± 2 μg/kg), located in Upper Silesia, and animals from STA had the lowest marginal mean Hg content in the liver (1.5 μg/kg). Differences between estimated marginal mean concentrations of Hg among study sites are presented in Supplementary Table S4 for clarification. Some differences between species were also salient. The levels of Hg in the livers of *A. flavicollis* were about half those of *A. agrarius* and *M. arvalis* (Figure 2A). The estimated mean Hg level in the livers of *M. glareolus* was 4.7 ± 2 μg/kg, which was almost twice that of *A. flavicollis*, but the difference was not confirmed statistically. Comparing the differences between sexes, it was found that males tended to accumulate about 38% more Hg in their livers than females (Figure 2B). Rodent body weight and Hg concentration in the liver were positively correlated, as shown by the Spearman rank correlation test (Figure 3), which confirmed the GLM findings.

## 3. Discussion

We found the highest levels of Hg in rodents from the DAB and GLW areas. The DAB area is located in the northeastern part of the Małopolskie voivodeship. According to national monitoring data, the median background Hg level in topsoil collected at the DAB site was 0.090 mg/kg (Supplementary Table S1). Surprisingly, there were no known major emitters close to the area, such as power plants, smelters, or other heavy industrial facilities, that may have been a source of Hg to the surrounding environment. We assume that local emissions caused by the combustion of coal in household boilers could also contribute to Hg bioaccumulation in biota [32]. The second region to the DAB site in terms of Hg content in the livers of rodents was located in the western part of the Upper Silesian Industrial District, which is known as one of the most polluted parts of Poland and is mainly associated with coal mining and metal production. Mercury levels in this area could be three- to sixfold higher than in rural areas in Poland [33], which our findings confirm.

The mean concentration of Hg found in the livers of all species of rodents from the most polluted DAB site (15 µg/kg of wet weight) was one-seventh of the level of Hg found in the livers of *A. flavicollis* from areas polluted by lead smelting in Slovenia, but threefold higher than that observed in the same species captured in the area contaminated by power plant emissions in that country [34]. Much higher levels than ours were found in *Microtus guentheri* from the marble mining area in Turkey [35], in *M. glareolus* from the zone around a chlor-alkali plant in Great Britain [36], and in *Apodemus sylvaticus* from different polluted and unpolluted areas in Galicia in northern Spain [37]. However, threefold lower Hg levels were noted in *M. glareolus* inhabiting the area affected by metal-processing industry in Russia [18]. Literature data on Hg levels in the livers of wild rodents are summarized in Table 1.

Mercury can accumulate along the trophic gradient in food webs, and the level of this element increases with the higher trophic position of animals [3,18]. However, the bioaccumulation of Hg may depend on dietary protein level and glutathione metabolism [38] and on the role of gut microbiota in demethylation and excretion of Hg [39]. The lowest liver Hg levels were found in *A. flavicollis*. Our results are in line with the results of Martiniaková et al. [40], who found that *A. flavicollis* was a biomonitor with lower metal concentration than *M. glareolus*. Mice of the *Apodemus* species have a more variable and protein-rich diet than herbivorous voles [41], and the richness of diet may result in lower bioaccumulation of toxic elements due to “diet dilution” [42]. We hypothesize that mycophagy could be another explanation for species-specific differences in liver Hg concentration in rodents. Fungi can accumulate Hg from the environment, and Hg levels in fruiting bodies could be higher than 4 mg/kg of dry weight [43,44,45]. In our study, all rodents were captured from early summer to the end of October, when fungi were readily available. Blaschke and Bäumler reported that fungal spores can account for 7% of the stomach volume of *A. flavicollis* and up to 36% of the stomach content of *M. glareolus* [46]. Besides species-specific feeding habits, the frequency of mycophagy in rodents also depends on the availability of this dietary component during the year. The analysis of fungal spores in fresh fecal pellets of rodents showed that they were present in almost 100% of examined individuals of *M. glareolus* during summer and autumn, whereas in *Apodemus* spp., only 30–40% of individuals consumed fungi during summer, although the frequency increased to approximately 80% in autumn [47]. The bioaccumulation of Hg may be different between the sexes. Lower levels of Hg in female mammals may be due to depuration during lactation [48].

The higher Hg concentrations in the livers of rodents found in our study corroborate the results reported by Sánchez-Chardi in white-toothed shrews (*Crocidura russula*) [52]. We are aware that the lack of the ages of the rodents, whch were not recorded during the study, is a limitation of our research. The estimation of the age of rodents by body size could be imprecise because different factors affect their growth [53]. Nevertheless, we found a positive correlation between body weight and liver Hg in rodents, which could be explained by the accumulation of Hg within their lifespan. Our study showed that the concentration of Hg in the liver of wild rodents may depend on different factors, including the level of exposure in their habitat, species, sex, and b.w. We suspect that differences in liver Hg concentrations between species of rodents may be caused by feeding habits, and future studies are needed to investigate the potential sources of Hg in their diet. 

## 4. Materials and Methods

### 4.1. Sampling

A total of 221 free-living small rodents were captured between June 2016 and October 2017 in 12 study sites (counties) of central, southeastern, and eastern Poland. The characteristics of all study sites, including mean annual temperature, annual rainfall, altitude, type of vegetation, and background soil Hg, and the number of animals sampled per species and sex are summarized in Supplementary Table S1. All animals were caught by a standard live-trapping technique in their natural foraging areas located close to farm buildings, using Sherman traps with baits of cereal grain and fresh apple. Four species of wild rodents were chosen: the bank vole (*M. glareolus*), common vole (*M. arvalis*), striped field mouse (*A. agrarius*), and yellow-necked mouse (*A. flavicollis*). Live animals were transported to the laboratory and euthanized, and necropsies were performed in a laminar chamber on the same day as capture. Liver samples were taken using stainless-steel surgical scissors, placed in 1.5 mL Eppendorf tubes, and frozen at −20 °C until analysis. The sampling of rodents was performed within a project that was focused on small mammals as sentinels for multiple zoonotic pathogens and was approved by the Local Ethics Committee for Animal Experimentation in Lublin under Resolution No. 30/2016. No ethical committee permission was required for the analysis of Hg, as the samples were taken post-mortem for this purpose.

### 4.2. Mercury Analysis

Frozen samples were thawed at 4 °C. The analysis of Hg in liver tissue was performed in raw tissue by a previously described method [54] using a Tri-cell DMA-80^®^ direct mercury analyzer (Milestone Srl, Sorisole (BG), Italy). Quantification of Hg was based on external calibration curves in three independent working ranges. Standard solutions (from 3 to 30, from 3 to 150, and from 250 to 10,000 µg/L) were prepared by dilution of an Hg standard stock solution (J.T. Baker, 1000 mg/L) (Avantor Performance Materials B.V, Deventer, the Netherlands) with 1% (*v*/*v*) nitric acid (Suprapur^®^, Merck, Darmstadt, Germany). Briefly, using an ENTRIS 224l-1S analytical balance (Sartorius Lab Instruments GmbH & Co, Goettingen, Germany) approximately 50 ± 0.1 mg of liver tissue was weighed into nickel boats and placed on the autosampler rotor of the DMA-80. The analysis took 5.5 min per sample. The operating conditions are shown in Supplementary Table S2. Quality control of the measurements was provided by using the following certified reference materials (CRMs): SRM-1577c Bovine Liver (National Institute of Standards and Technology (NIST), Gaithersburg, MD, USA), Chicken ZC73016 (NCS Testing Technology Co., Beijing, China), and MODAS-3 Herring tissue (M-3 HerTis) (Institute of Nuclear and Technology (IChTJ), Warsaw, Poland). Recoveries of Hg in CRMs were 98%, 122%, and 97% for SRM-1577c, ZC73016, and M-3 HerTis, respectively. The limit of quantification of the method was 1 µg/kg of wet weight. The method is accredited according to ISO/IEC 17025/Ap1:2007 [55] and regularly verified in proficiency tests organized by the European Union Reference Laboratory for Metals and Nitrogenous Compounds in Feed and Food (EURL-MN) in Lyngby, Denmark.

We also measured the moisture content in the livers of rodents to facilitate the comparison of our results with literature data. The moisture content was analyzed in 16 randomly selected subsamples of the liver using an HR83 moisture analyzer (Mettler Toledo, Switzerland) according to the manufacturer’s instructions. The mean content of dry matter in the liver of rodents was 30.4%, and this level was used for calculations. All of the results in this article are expressed in µg/kg of wet weight.

### 4.3. Statistical Analysis

Statistical analysis was performed using R in version 3.6.0 [56]. The data handling and descriptive statistics calculation, including mean, standard deviation, median, median absolute deviation (MAD), and range were performed in the dplyr package, version 0.8.1 [57]. Results below LOQ were set as 0.5 of the LOQ. The Shapiro–Wilk normality test was used to verify the normality of distribution [58], and because data were not normally distributed, we used log-transformation to achieve the normality. Effects of species, study site, sex, season of sampling (summer and autumn), feeding habits (omnivores and herbivores), and body weight were verified using GLM [59]. We constructed GLM with Gaussian distribution as follows: log-transformed Hg concentration was used as the dependent variable, and all factors (species, study site, sex, season of sampling, feeding habit, and body weight) were used as predictors. The best model was chosen by the step command with forward–backward stepwise procedure based on Akaïke’s Information Criterion (AIC). Differences between factor levels were verified by post-hoc tests with Bonferroni adjustment of *p*-values on estimated marginal means using version 1.4.1 of the emmeans package [60]. The Spearman’s rank correlation coefficient was used to verify the relationship between Hg accumulation in the liver and the b.w. The results were visualized using the ggplot2 package (version 3.2.1) [61], and the map with estimated marginal mean concentrations of Hg in the livers of rodents in selected study sites was plotted by QGIS software version 3.8 [62].

## Figures and Tables

**Table 1:** Concentrations of Hg in the livers of small terrestrial mammals from different areas (in µg/kg of wet weight).

Species	Country	Site	*N*	Mean (Min–Max)	Reference
*Apodemus flavicollis*	Slovenia	lead smelter	7	100.3 (39.5–249.3) *	[34]
		main road	23	6.1 (3–21.3) *	
		thermal power plant	30	42.6 (3–173.3) *	
		reference area	13	18.2 (3–36.5) *	
*Apodemu sylvaticus*	Great Britain	chlor-alkali plant	6	230 (90–530)	[36]
		reference area	10	40 (10–70)	
	Spain	Galicia, different areas	372	53 (17–110)	[37]
*Chaetidypus penicillatus*	NV, USA	Las Vegas Wash	32	3.3 (0.9–24.3)	[49]
*Dipodomys merriami*			8	3.7 (0.7–20.6)	
*Microtus arvalis*	Slovenia	thermal power plant	4	3 (<LOQ–10) *	[34]
		main road	3	3 *	
*Microtus guentheri*	Turkey	marble mining area	68	231 (221.9–240.1) *	[35]
		reference area	24	200.6 (145.9–240.1) *	
*Mus musculus*	NV, USA	Las Vegas Wash	2	2.3 (1.5–3.0)	[49]
*Myodes glareolus*	Slovenia	lead smelter	21	9.1*	[34]
		main road	13	3 (3–6.1) *	
		thermal power plant	4	97.3 (3–231) *	
		reference area	15	18.2 (3–36.5) *	
	Russia	metallurgical plant	50	4.3 *	[18]
	Great Britain	chlor-alkali plant	7	150 (60–340)	[36]
		reference area	6	60 (30–130)	
*Neotoma lepida*	NV, USA	Las Vegas Wash	16	6.8 (2.0–20.8)	[49]
*Peromyscus leucopus*	IL, USA	contaminated wetland	36	11 (2–23)	[50]
		reference area 1	84	10 (1–21)	
		reference area 2	43	8 (1–20)	
		reference area 3	43	15 (3–35)	
*Peromyscus maniculatus*	MI, USA	outside Sargent Lake watershed	15	29.98	[51]
		inside Sargent Lake watershed	15	10.99	
		mainland	4	21.41	
*Peromyscus eremicus*	NV, USA	Las Vegas Wash	46	10.9 (0.9–85.3)	[49]

* Results calculated from original data given on dry wt. basis assuming 30.4% of solids.

## Supplementary Materials

The following are available online. Supplementary Table S1 of the characteristics of study sites and the number of rodents captured according to study site, species, and sex; Supplementary Table S2 of the DMA-80 operating conditions; Supplementary Table S3 of the descriptive statistics of Hg concentrations in the liver of rodents according to their species, sampling site, and sex (µg/kg); Supplementary Table S4 of the differences in liver Hg concentrations in rodents between study sites; Supplementary Table S5 of the differences in liver Hg concentrations in rodents between species; and Supplementary Table S6 of the differences in liver Hg concentrations in rodents between females and males.
