# Supplementary material for: Concentration of Mercury in the Livers of Small Terrestrial Rodents from Rural Areas in Poland
Source: Molecules. 2019 Nov 14;24(22):4108. doi: 10.3390/molecules24224108 (PMC6891690; doi:10.3390/molecules24224108)
Supplement: Supplementary file 1 [file molecules-24-04108-s001.pdf]

molecules

Supplementary Materials

Concentration of Mercury in the Liver of Small Terrestrial Rodents from Rural Areas in Poland

Maciej Durkalec [1,*], Agnieszka Nawrocka [1], Jacek Żmudzki [2], Aleksandra Filipek [1], Marcin Niemcewicz [3] and Andrzej Posyniak [1]

1. Department of Pharmacology and Toxicology, National Veterinary Research Institute, Aleja Partyzantow 57, 24-100 Puławy, Poland; agnieszka.nawrocka@piwet.pulawy.pl (A.N.); aleksandra.filipek@piwet.pulawy.pl (A.F.); aposyn@piwet.pulawy.pl (A.P.)
2. Department of Swine Diseases; National Veterinary Research Institute, Aleja Partyzantow 57, 24-100 Puławy, Poland; jaca@piwet.pulawy.pl
3. Biological Threats Identification and Countermeasure Centre, Military Institute of Hygiene and Epidemiology, Lubelska 2, 24-100 Puławy, Poland; marcinniem@wihe.pulawy.pl

* Correspondence: maciej.durkalec@piwet.pulawy.pl; Tel.: +48-81-889-3165

Supplementary Table S1. The characteristics of study sites and the number of rodents captured by county, species, and sex.

County	Code	Altitude, m a. s. l.	Mean Annual Temerature, °C	Mean Annual Rainfall, mm H_2O/m^2	Background Soil Hg [1] Median (Range), in mg/kg	Vegetation [2]	N	Species		Sex	
Dąbrowski (Dąbrowa Tarnowska)	DAB	201	8.4	668	0.09 (0.07–0.20)	*Tilio-carpinetum*	*12*	*A. agrarius*	7	F	6
										M	1
								A. flavicollis	4	F	1
										M	3
								M. arvalis	1	F	1
Gliwicki	GLW	235	8.5	663	0.05 (0.05–0.26)	*Tilio-carpinetum*	*13*	*A. agrarius*	2	F	2
								A. flavicollis	9	F	6
										M	3
								M. arvalis	2	F	2
Lubliniecki	LUB	264	8.3	633	<0.05 (<0.05–0.07)	*Tilio-carpinetum*	*8*	*A. flavicollis*	3	F	3
								M. arvalis	5	F	3
										M	2
Niżański	NIS	168	7.8	572	0.05 (<0.05–0.09)	*Querco-pinetum*	*20*	*A. agrarius*	14	F	8
										M	6
								A. flavicollis	4	F	3
										M	1
								M. arvalis	2	M	2
Oświęcimski	OSW	248	8.6	719	0.07 (0.05–0.74)	*Tilio-carpinetum*	*24*	*A. agrarius*	11	F	5
										M	6
								A. flavicollis	10	F	4
										M	6
								M. arvalis	3	F	2
										M	1
Przemyski	PRZ	215	7.9	634	0.10 (0.07–0.18)	*Dentario glandulosae-Fagetum*	*20*	*A. agrarius*	5	F	2
										M	3
								A. flavicollis	9	F	2
										M	7
								M. arvalis	6	F	3
										M	3

Rawski	RAW	142	7.7	542	<0.05 (<0.05–0.07)	*Potentillo albae-Quercetum*	*24*	*A. agrarius*	8	F	6
										M	2
								A. flavicollis	13	F	9
										M	4
								M. arvalis	3	M	3
Sieradzki	SRD	129	7.5	608	<0.05 (<0.05–0.07)	*Querco-pinetum*	*23*	*A. agrarius*	7	F	7
								A. flavicollis	13	F	9
										M	4
								M. arvalis	3	F	1
										M	2
Starachowicki	STA	275	7.2	598	<0.05 (<0.05–0.08)	*Calamagrostio-Quercetum*	*17*	*A. agrarius*	3	F	2
										M	1
								A. flavicollis	13	F	7
										M	6
								M. arvalis	1	M	1
Świdnicki	SWI	232	7.4	544	<0.05 (<0.05–0.11)	*Tilio-carpinetum*	*17*	*A. flavicollis*	13	F	5
										M	8
								M. arvalis	2	F	2
								M. glareolus	2	F	1
										M	1
Tomaszowski	TOM	294	7.3	590	<0.06 (<0.05–0.07)	*Querco-pinetum*	*29*	*A. agrarius*	6	F	1
										M	5
								A. flavicollis	20	F	13
										M	7
								M. arvalis	2	F	1
										M	1
								M. glareolus	1	F	1
Włoszczowski	WLO	245	7.5	626	<0.05 (<0.05–0.05)	*Querco-pinetum*	*14*	*A. agrarius*	7	F	4
										M	3
								A. flavicollis	7	F	3
										M	4

[1] Data was extracted from the Central Geological Database of the Polish Geological Institue—National Research Institute, available at: http://baza.pgi.gov.pl. [2] Types of vegetation were taken from the map of Potential natural vegetation of Poland available at: https://www.igipz.pan.pl/potential-vegetation-zgik.html.

Supplementary Table S2. DMA-80 operating conditions.

Step	Time (min)	Temperature (°C)
1	1	20–200
2	1.5	200–650
3	1.5	650
4	1.5	650

Supplementary Table S3. Descriptive statistics of Hg concentrations in the liver of rodents according to their species, sampling site, and sex (μg/kg).

	N	mean	SD	median	MAD	min	max	mean	SD	median	MAD	min	max
		**Expressed on Dry Weight Basis**						**Expressed on Wet Weight Basis**					
**Species**													
A. agrarius	70	18.1	20	11.6	8	1.6	119.7	5.5	6	3.5	2	<LOQ	36.4
A. flavicollis	118	14.6	16	10.4	9	1.6	97.0	4.4	5	3.2	3	<LOQ	29.5
M. arvalis	30	15.4	12	14.0	7	1.6	52.6	4.7	4	4.3	2	<LOQ	16.0
M. glareolus	3	35.2	33	30.4	38	4.9	70.2	10.7	10	9.2	12	1.5	21.4
**Study site**													
DAB	12	48.0	33	40.9	25	10.2	119.7	14.6	10	12.5	8	3.1	36.4
GLW	13	22.7	19	18.7	11	6.2	72.0	6.9	6	5.7	3	1.9	21.9
LUB	8	6.5	4	6.2	5	2.3	11.2	2.0	1	1.9	2	<LOQ	3.4
NIS	20	10.0	7	8.4	4	2.6	28.6	3.0	2	2.6	1	<LOQ	8.7
OSW	24	11.1	10	7.7	5	3.3	52.6	3.4	3	2.4	2	1.0	16.0
PRZ	20	9.3	7	6.7	5	1.6	24.3	2.8	2	2.1	2	<LOQ	7.4
RAW	24	12.5	8	10.2	7	3.3	31.6	3.8	2	3.1	2	1.0	9.6
SRD	23	10.8	8	9.5	7	2.3	39.4	3.3	2	2.9	2	<LOQ	12.0
STA	17	5.0	4	3.9	3	1.6	16.1	1.5	1	1.2	1	<LOQ	4.9
SWI	17	28.4	23	17.5	19	1.6	74.5	8.6	7	5.3	6	<LOQ	22.7
TOM	29	19.6	14	13.3	12	3.8	52.4	5.9	4	4.0	4	1.2	15.9
WLO	14	20.9	21	14.1	8	1.6	66.7	6.4	6	4.3	2	<LOQ	20.3
**Sex**													
Female	125	13.8	13	10.2	8	1.6	72.0	4.2	4	3.1	3	<LOQ	21.9
Male	96	19.0	21	12.1	10	1.6	119.7	5.8	6	3.7	3	<LOQ	36.4

Supplementary Table S4. Differences in liver Hg concentrations in rodents between study sites. The results are expressed as marginal means with standard error and confidence intervals calculated based on the GLM model and averaged by species and sex, and back-transformed from the log scale (in µg/kg of wet weight). Differences between estimated marginal means were verified using the Tukey test on log scale values.

County	Mean ± SE	CI	*p*-value											
			DAB	GLW	LUB	NIS	OSW	PRZ	RAW	SRD	STA	SWI	TOM	WLO
DAB	15 ± 4	9.3–24.3	-	ns	<0.0001	<0.0001	<0.0001	<0.0001	<0.0001	<0.0001	<0.0001	ns	<0.01	<0.01
GLW	7.9 ± 2	5–12.6	ns	-	<0.01	<0.01	<0.01	<0.001	ns	ns	<0.0001	ns	ns	ns
LUB	2.2 ± 1	1.2–3.8	<0.0001	<0.01	-	ns	ns	ns	ns	ns	ns	ns	ns	ns
NIS	2.7 ± 1	1.8–4	<0.0001	<0.01	ns	-	ns	ns	ns	ns	ns	ns	ns	ns
OSW	3.3 ± 1	2.2–4.8	<0.0001	<0.01	ns	ns	-	ns	ns	ns	<0.05	ns	ns	ns
PRZ	2.4 ± 1	1.6–3.5	<0.0001	<0.001	ns	ns	ns	-	ns	ns	ns	<0.05	ns	ns
RAW	3.8 ± 1	2.7–5.5	<0.0001	ns	ns	ns	ns	ns	-	ns	<0.01	ns	ns	ns
SRD	3.7 ± 1	2.5–5.4	<0.0001	ns	ns	ns	ns	ns	ns	-	<0.01	ns	ns	ns
STA	1.5 ± 0	1–2.3	<0.0001	<0.0001	ns	ns	<0.05	ns	<0.01	<0.01	-	<0.0001	<0.0001	<0.0001
SWI	6.1 ± 1	4.2–9	ns	ns	ns	ns	ns	<0.05	ns	ns	<0.0001	-	ns	ns
TOM	4.9 ± 1	3.4–6.8	<0.01	ns	ns	ns	ns	ns	ns	ns	<0.0001	ns	-	ns
WLO	4.9 ± 1	3.1–7.6	<0.01	ns	ns	ns	ns	ns	ns	ns	<0.0001	ns	ns	-

ns—not siginificant.

Supplementary Table S5. Differences in liver Hg concentrations in rodents between species of rodents. The results are expressed as marginal means with standard error and confidence intervals calculated based on the GLM model and averaged by study site and sex, and back-transformed from the log scale (in µg/kg of wet weight). Differences between estimated marginal means were verified using the Tukey test on log scale values.

Species	Mean ± SE	CI	p-value			
			A. agrarius	*A. flavicollis*	*M. arvalis*	*M. glareolus*
A. agrarius	4.3 ± 0	3.5–5.3	-	0.0022	ns	ns
A. flavicollis	2.7 ± 0	2.3–3.1	0.0022	-	0.0045	ns
M. arvalis	4.6 ± 1	3.6–6.2	ns	0.0045	-	ns
M. glareolus	4.7 ± 2	2–11.2	ns	ns	ns	-

ns—not siginificant.

Supplementary Table S6. Differences in liver Hg concentrations in rodents between females and males. The results are expressed as marginal means with standard error and confidence intervals calculated based on the GLM model and averaged by site and species, and back-transformed from the log scale (in µg/kg of wet wt.). Differences between estimated marginal means were verified using the Tukey test on log scale values.

**Sex**	**Mean ± SE**	**CI**	***p*-value**	
			**Females**	**Males**
Females	3.4 ± 0	2.7–4.4	-	0.0017
Males	4.7 ± 1	3.6–6.1	0.0017	-
